# Emerging strategies for controlled digestion of fat substitutes: Synergistic modification of egg white protein by combined polyphenol heat treatment for preparation of double network emulsion gel^☆^

**DOI:** 10.1016/j.fochx.2025.102784

**Published:** 2025-07-12

**Authors:** Renzhao Zhang, Jingbo Liu, Meijing Yu, Qiri Mu, Zhaohui Yan, Yudan Zhang, Yutong Zhang, Ting Zhang, Xuanting Liu

**Affiliations:** Jilin Provincial Key Laboratory of Nutrition and Functional Food and College of Food Science and Engineering, Jilin University, Changchun 130062, China

**Keywords:** Egg white protein, Emulsion gel, In vitro digestion, Microstructure, Fat substitute

## Abstract

This study investigated the differential effects of polyphenol-thermal synergistic modification on egg white protein in double network emulsion gels (DNEG), which endowed them with digestion-controllable properties as fat substitutes. Thermal treatment induced protein conformational unfolding and promoted polyphenol-mediated gel network refinement. Microstructural analysis revealed that DNEG progressively formed a homogeneous framework, thereby enhancing water-holding capacity and gel strength to 56.1 % and 353.3 g, respectively. The refined gel structure delayed digestive enzyme diffusion, resulting in a 60.5 % increase in vitamin D_3_ release compared to the control. Furthermore, DNEG exhibited sensory attributes comparable to full-fat counterparts in low-fat meat products, which offered novel insights for engineering functional fat substitutes.

## Highlights

• PC and heat treatment synergistically refined the DNEG structure.
• The delicate network structure of DNEG could inhibit digestive enzymolysis.
• Enhancing the fat-substitution capacity of DNEG by dual modification of EWP.
• Adjustment of the DNEG structure regulated the level of DNEG digestion.

## Graphical abstract

**Figure:**
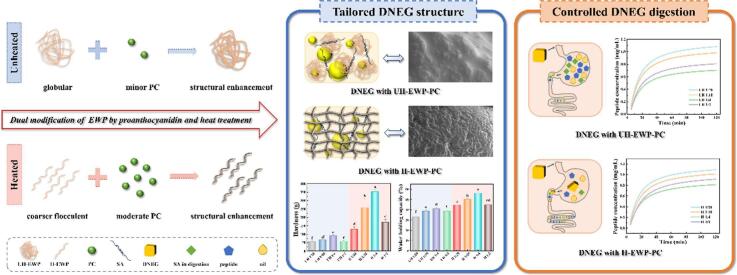

## 1 Introduction

As indispensable functional components in food systems, dietary fats play pivotal roles in improving food texture, delivering flavor compounds, and maintaining energy balance (Yen et al., 2023). Animal-derived fats such as pork back fat have been widely utilized in meat products, baked goods, and traditional dishes due to their unique fatty acid composition (e.g., triglycerides, cholesterol) and superior physical properties (Badar et al., 2023). However, with evolving dietary patterns and advancements in food processing technologies, traditional animal fats now face dual challenges. On one hand, excessive consumption of pork back fat may elevate the risks of cardiovascular diseases and hypertension (Lee et al., 2025). On the other hand, their use contributes to increased carbon footprints in livestock farming and higher water consumption, posing challenges to environmental sustainability (Zembyla et al., 2020). Addressing these contradictions has driven an urgent need to develop novel fat substitutes in food science.

In recent years, advancements in food technologies have spurred the emergence of innovative fat substitutes, gradually applied in low-fat meat sausages, reduced-fat cream cakes, and other health-oriented products (Yan, Liu, Cao, et al., 2023; Zhang et al., 2024). For instance, inulin had been commercially adopted in yogurts and butter due to its gel-forming properties, smooth texture, and viscous mouthfeel (Li et al., 2022). However, its limited digestibility, potential gastrointestinal discomfort upon prolonged intake, and inability to fully mimic fat's nutritional functions (e.g., facilitating fat-soluble vitamin absorption) restricted its applications (Yang et al., 2020). Protein-based substitutes, including enzymatically hydrolyzed proteins, had gained traction owing to their emulsifying capacity, nutritional benefits, and safety. Nevertheless, their stability during digestion was highly sensitive to environmental factors like pH and temperature, limiting their applicability and consumer acceptance (Y. Lu et al., 2021). Thus, understanding the digestive behavior of fat substitutes was critical for tailoring low-fat, health-focused foods. Developing substitutes that mimic fat's structural and sensory qualities while enabling controlled digestion was paramount for the food industry.

Egg white protein (EWP) has high nutritional value and contains a complete amino acid profile. This provides it with high methionine content, excellent stability, low cost-effectiveness, and a simple production method, which attracts widespread attention from the scientific community and the business community as an ideal raw material for emulsified gel-type fat substitutes (Zang et al., 2023). Unlike other emulsion gels focusing on gelation method innovations, protein-based systems prioritized the physicochemical state of raw materials, as this directly dictates gel quality (Zembyla et al., 2020). Common modification strategies such as pH-shifting, freeze-thaw cycling, and thermal treatment often involve harsh protein alterations, necessitating gentler modification approaches (Grasberger et al., 2024). Recent studies have increasingly explored small-molecule modifiers like polyphenols. For example, Shu et al. (2025) investigated rice protein-polyphenol interactions, which demonstrated that gallic acid-enhanced interfacial structures inhibited lipid oxidation. Mohammadi et al. (2023) achieved plant polyphenol-grafted soybean protein assemblies for stable 3D-printable emulsion gel inks. Similarly, our prior work revealed that phenolic hydroxyl-modified EWP imparted exceptional flexibility to double network emulsion gel (DNEG), enhancing oral tribological and wettability properties (Zhang et al., 2025). However, single-factor modification treatment had limited effectiveness in improving the processing characteristics of proteins. Some researchers insisted that single acid treatment could improve the functional characteristics of soy protein isolate, but it was less effective than single or combined pepsin hydrolysis and high-pressure homogenization under acidic conditions. However, some researchers still believe that single-factor modification treatments have limited effectiveness in improving protein processing characteristics. For example, single-enzyme modification treatments had poor efficacy in improving protein properties (Yuan et al., 2012). Therefore, the use of a dual modification method combining phlorotannins with enzymatic hydrolysis to modify soy protein isolates not only enhanced enzymatic hydrolysis efficiency but also improved the functional properties of protein complexes (Lian et al., 2025). Therefore, combined processing would be particularly important for protein modification, especially the synergistic effect of thermal processing with small molecules such as polyphenols. This approach might not only meet the demand for reducing animal fat intake through emulsion gel fat substitutes in health meat foods, but also enable controlled lipidic digestion, thereby serving a dual purpose. Hence, it would be imperative to clarify the structural characteristics, digestive characteristics, and functional characteristics of EWP-based emulsion gel-type fat substitutes.

To achieve pork back fat substitution, this study thermally pretreated egg white protein (EWP) synergistically modified with proanthocyanidins (PC), then constructed a double network emulsion gel (DNEG) using sodium alginate (SA). Molecular docking and spectroscopic techniques were utilized to elucidate the structural impacts of binary synergistic modification on EWP. The mechanisms by which protein structural changes influence emulsification and interfacial behaviors were systematically evaluated. The fat-mimetic potential of DNEG was comprehensively assessed, spanning microstructural networks to macro-textural properties. In addition, digestive behaviors were scrutinized to analyze how thermal treatment and PC dosage modulated limited digestion and controlled VD_3_ release. Collectively, this work would provide innovative strategies for developing low-fat health foods with enhanced functionalities and precision nutrition regulation.

## 2 Materials and methods

### 2.1 Materials and reagents

Egg white protein (EWP, protein ≥90 % *w*/w) was supplied by Ovodan Foods Co., Ltd. (Jiangsu, China). Proanthocyanidin (PC) was sourced from Yuanye Chemical Co., Ltd. (Shanghai, China). Glucono – δ – lactone (GDL) was acquired from Sigma Aldrich Chemical Co. (Missouri, USA). Soybean oil, fresh lean pork, pork back fat, and other food-grade ingredients were sourced from OUYA Supermarket (Jilin, China). All other reagents (of at least analytical grade) were purchased from Sinopharm Chemical Reagent Co., Ltd. (Shanghai, China).

### 2.2 Preparation of double network emulsion gel (DNEG)

Light modifications to the methodology based on previous work (Zhang et al., 2025). Accurately weighed EWP powder was placed in ultrapure water and stirred (25 °C, 500 rpm, 2 h) to obtain an 8 wt% EWP dispersion. The pH of the EWP dispersion was adjusted to 7.0 with 1.0 M HCl or NaOH solution and then left in the freezer (4 °C, 12 h) to complete hydration. The following day, EWP dispersion was divided into two equal portions. One of the portions was subjected to a thermostatic water bath (50 °C, 20 min), after which the ice water bath was cooled to room temperature and called H-EWP. Another copy without heat treatment was called UH-EWP. Based on the results of the preliminary experiment, the experimental method described in the previously published work were modified (Wen et al., 2023). Different amounts of PC were added to H-EWP, UH-EWP to achieve the mass ratio of polyphenols and proteins (1:2, 1:4, 1:10, 1:20). Then stir at room temperature away from light (500 rpm, 30 min) to ensure they thoroughly combined. Subsequently, food-grade soybean oil and different EWP were sequentially overmixed at a 2:3 ratio by volume and immediately subjected to high-speed homogenization (12,000 rpm, 3 min) to obtain the primary emulsion. The GDL (0.18 g/g protein), 0.3 wt% sodium alginate (SA), and Ca^2+^ (20 mM) were added to the prepared EWP emulsion. And the emulsion was homogenized for 1 min at 6000 rpm using a THF500-18G homogenizer (Tuohe, Shanghai, China). Finally, they were left at 50 °C for 2 h to form DNEG. Freshly formed samples were allowed to fully mature after standing (4 °C, 12 h) in the freezer for further analysis.

Only when determining the carrying stability of DNEG in the digestive experiment, the vitamin D_3_ was added to the oil at a concentration of 250 mg/mL prior.

### 2.3 Molecular docking

This study was based on structural biology database resources to obtain three-dimensional conformational information of the target molecules (Yan et al., 2025). The crystal structure of ovalbumin (OVA) was obtained from the RCSB Protein Data Bank (PDB ID: 1UHG), and the molecular conformation of PC was taken from PubChem Compound Database (PubChem CID: 108065). The molecular docking simulation methodology was used to construct the study system by creating a cubic grid box with its geometric center located in the Cartesian coordinate system. The spatial configuration of the OVA-PC complex was modeled and characterized in three dimensions with the help of PyMOL v2.2.0 molecular visualization platform. The interface of the complex was quantitatively analyzed using the Discovery Studio Visualizer 2024 program, and the intermolecular interaction modes, such as hydrogen bonding network and hydrophobic interactions, were systematically analyzed, and the key ligand-receptor binding sites were accurately labeled.

### 2.4 Average particle size and zeta-potential

The average particle size and the zeta potential of all primary emulsion samples were measured at room temperature employing a Zeta sizer Nano ZS90 particle size analyzer (Malvern, Worcestershire, UK). The refractive indices of the materials and solvents were 1.45 and 1.33, respectively. The samples were diluted with PBS (10 mM, pH 7) at room temperature at a ratio of 1:200 to avoid multiple scattering. Each measurement was run at least 5 times (Yan, Liu, Li, et al., 2023).

### 2.5 Emulsifying activity index (EAI)and emulsion stability index (ESI)

After emulsion preparation, 100 μL of sample was quickly pipetted and vortex-mixed with 5 mL of 0.1 % SDS solution. The initial absorbance value was measured at 500 nm using UV spectrophotometry. Subsequently, the emulsion system was allowed to stand for 40 min, and then 100 μL of sample was accurately pipetted to repeat the test. Finally, the EAI and ESI were calculated by eqs. (1), (2), respectively (Yan, Liu, Ren, et al., 2023).

(1) $$\mathrm{EAI}=\frac{2\times 2.303}{C\times \left(1-\varphi \right)\times 10^{4}}\times A_{0}\times N$$

(2) $$\mathrm{ESI}\;\left(\min\right)=\frac{A_{30}\times \Delta T}{A_{0}-A_{30}}\times 100\%$$

where A_0_ and A_30_ meant the absorbance at 0 and 30 min. C was the concentration of protein solution (g/mL). N was the dilution factor, and Ф referred to the volume fraction of oil, and ΔT was 30 min.

### 2.6 Dynamic surface tension

The determination of dynamic interfacial tension was based on a slight modification of the previous method (Yan et al., 2025). Changes in tension at the air-water interface were dynamically evaluated for different samples using a DSA25 interfacial analyzer (KRÜSS, Hamburg, Germany). To evaluate the interfacial tension, a 10 μL droplet of emulsion was generated at the injection needle, and the shape of the droplet was continuously recorded by a camera at a rate of one data point per second for 250 s. Dynamic interfacial tension values were calculated for each sample using the Laplace-Young equation analysis based on the droplet shape recorded by a high-speed camera.

### 2.7 Texture profile analysis (TPA)

The TPA test was evaluated concerning the modified method of Zhang et al. (2024). Samples were cut into squares (20 mm per side) at room temperature (25 °C). DNEG was placed on a CT3-4500 g texture analyzer (Brookfield, MA, USA), and properties such as gel hardness were determined by compression of 50 % using a TA-5 probe at 1.0 mm/s in TPA mode. At least three replicates of each experiment were required.

### 2.8 Water holding capacity (WHC)

The determination of WHC content in the samples was based on published methods (Zhang et al., 2025). A qualitative filter paper with a diameter of 12.5 cm was used to accurately weigh 3.0 g of DNEG, and the filter paper and the sample were placed together in a 50 mL round-bottomed centrifuge tube and centrifuged in a low-temperature, high-speed centrifuge with the following parameters (4 °C, 3500 g, 10 min). Based on the change in gel mass before and after centrifugation, the WHC of the sample was calculated using the following eq. (3):

(3) $$\mathrm{WHC}\;\left(\%\right)=\frac{M1}{M0}\times 100$$

where M_0_ and M_1_ represented the weight of the sample before and after centrifugation, respectively.

### 2.9 Intermolecular forces of DNEG

The 0.5 g sample was accurately weighed and mixed with L1, L2, L3 and L4 buffers (L1: 0.6 M NaCl; L2: 0.6 M NaCl with 1.5 M urea; L3: 0.6 M NaCl with 8 M urea; L4: 1 M NaOH). The mixture was then homogenized immediately (10,000 rpm for 30 s). The mixture was placed in a 50 mL centrifuge tube and centrifuged in a low-temperature high-speed centrifuge (4 °C, 10000 *g*, 10 min) to remove the precipitate. Finally, the supernatant was measured using a UV-2550 UV spectrophotometer (Shimadzu, Kyoto, Japan) at 595 nm. The actual percentage of intermolecular forces, such as ionic bonding, needs to be calculated by the following formulas (Zhang et al., 2025).

(4) $$\text{Ionic bond}\;\left(\%\right)=\frac{C_{1}}{C_{4}}\times 100$$

(5) $$\text{Hydrogen bond}\;\left(\%\right)=\frac{\left(C_{2}-C_{1}\right)}{C_{4}}\times 100$$

(6) $$\text{Hydrophobic interaction}\;\left(\%\right)=\frac{\left(C_{3}-C_{2}\right)}{C_{4}}\times 100$$

(7) $$\text{Covalent bonds}\;\left(\%\right)=\frac{\left(C_{4}-C_{3}\right)}{C_{4}}\times 100$$

where C represents the concentration of soluble proteins in the supernatant of L1-L4. The numerical relationships correspond one to one.

### 2.10 Fourier transform infrared spectroscopy (FT-IR)

Based on our previous experimental approach (Yan, Liu, Ren, et al., 2023), protein secondary structure measurements were performed using an iS20 FT-IR (Thermo Fisher Scientific, MA, USA) equipped with an ATR mode to identify functional group changes in response to the synergistic effect of heat treatment and polyphenols. Spectral recordings were analyzed in the range of 4000 to 1000 cm^−1^. The percentage of α-helix, β-folding, β-turning, and random curling in the protein secondary structure was calculated by Peak Fit software (v4.12). The experiments required all operations to be repeated at least 3 times.

### 2.11 Scanning electron microscopy (SEM)

The emulsion gels were cut into cubes (side length 1 cm) and then fixed in glutaraldehyde solution in a freezer at 4 °C for 12 h. After removal, the gels were rinsed three times with PBS to determine the removal of all fixative. The rinsed samples were sequentially eluted with 50 %, 70 %, 90 %, and 100 % ethanol solutions in a 10 min gradient. The samples were stored in tert-butanol in a refrigerator at −80 °C for 12 h before being placed in a low temperature freeze dryer for de-watering. The dried samples were adhered to conductive tape and placed on a sample stage. Regulus 8100 SEM (Hitachi, Tokyo, Japan) was applied to observe the microstructure of DNEG (Gao et al., 2024).

### 2.12 In vitro digestion

In order to investigate the effect of polyphenol synergistic heat treatment on the digestive properties of the fat substitute DNEG, the present study was carried out concerning the in vitro digestion model (Brodkorb et al., 2019; Liu et al., 2023; Yan, Liu, Ren, et al., 2023) for digestion simulation experiments. The simulated gastric fluid (SGF) and simulated intestinal fluid (SIF) master mixes were pre-prepared and refrigerated at 4 °C. A homogenizer was used to homogenize the DNEG samples to simulate the oral mastication process. In the simulated gastric digestion stage, 1.0 g of gel particles were accurately weighed and mixed with 20 mL of SGF, the pH of the system was adjusted to 2.0, and pepsin was added to a final viability of 2000 U/mL, and the coeliac was prepared. The digested system was incubated at 37 °C in a thermostatic shaker at 120 rpm for 2 h, and then the pH was adjusted to neutral to terminate the gastric digestion reaction.

In the intestinal digestion stage, the product of gastric digestion for 2 h was taken as substrate and mixed with simulated intestinal fluid at 1:1 (*v*/v). After adjusting the pH of the system to 7.0, trypsin (100 U/mL) was added, and a constant temperature oscillator was set at 37 °C with 12 times gradients (0, 5, 10, 15, 20, 30, 40, 50, 60, 80, 100, 120 min) from 0 to 120 min, and the oscillation frequency of 120 rpm was maintained to simulate the intestinal mechanical movement. After the digestion reaction was inactivated by boiling water bath for 2 min, it was immediately transferred to ice bath for 30 min to terminate the enzyme activity. The absorbance value was measured at 280 nm using a UV-2550 spectrophotometer (Shimadzu, Kyoto, Japan).

### 2.13 Bioavailability of vitamin D_3_ in DNEG

The 5 mL of sample was added to a 50 mL centrifuge tube containing 10 mL of ethanol in advance and vortexed for 10 s. The 10 mL of n-hexane was added to the mixture to extract and collect the organic layer. The mixed samples were centrifuged in a refrigerated centrifuge at 4000 *g* for 2 min to collect the upper extract. The above extraction was repeated three times to ensure that all the vitamin D_3_ was collected. The absorbance of the collected solution was measured at 265 nm and the loading of vitamin D_3_ was calculated according to eq. (8) (Ferreira et al., 2022).

(8) $$\text{Bioavailability of vitamin}\;D_{3}\;\left(\%\right)=\frac{C1}{C2}\times 100$$

where C_1_ and C_2_ represent the initial amount of vitamin D_3_ in the emulsion and the amount of vitamin D_3_ (μg/mL) in the emulsion after in vitro digestion treatment, respectively.

### 2.14 Digestion kinetics

The post-digested peptide concentration was determined with reference to cutting-edge methods (Liu et al., 2023). Specifically, DNEG samples after in vitro mock digestion were mixed with 20 wt% trichloroacetic acid solution (1:1, *V*/V), and the mixture was stored in a freezer at 4 °C. After 2 h, the mixture was centrifuged at 10000 *g* at 4 °C for 10 min. The amount of hydrolyzed peptides was determined by BCA immediately after the removal of the supernatant. The peptide concentrations at different time points in the gastric phase was fitted to Eq. (9), and the instantaneous digestibility was calculated from the derivative of Eq. (9) as shown in Eq. (10).

The first-order kinetic equation was used to fit the release curve of free amino groups during the digestion process. The y of each sample at various digestion times was fitted into Eq. (10). y_max_ was the final proteolysis extent at infinite digestion time, and the half-life time is the time needed to produce an amount of peptides equivalent to half y_max_.

(9) $$y=y_{\max}\times \exp\left(\frac{‐B}{\text{time}}\right)$$

where B =$\left(\text{half}‐\text{life time}\right)\times \ln2$.

(10) $$\frac{d_{y}}{d_{\mathrm{time}}}=60\times y_{\max}\times B\times \frac{1}{{\left(\text{time}\right)}^{2}}\times \exp\left(\frac{‐B}{\text{time}}\right)$$

where y_max_ was the degree of final protein hydrolysis, and the half-life time was the time required when 50 % of the peptide amount of y_max_ was produced.

### 2.15 Application of DNEG as fat substitutes

#### 2.15.1 Preparation of minced meat sausage

Lean pork was placed on ice to prevent it from spoiling, and the connective tissue and fat on top were promptly removed and processed for 1 min in high speed mode using a model S2-A808 meat grinder (Joyoung, Shandong, China) in mode to process the lean pork and pork back fat or fat substitute in high speed mode (Zhang et al., 2024). Mix pork lean meat and fat (or DNEG) at a ratio of 3:1. Co-ingredients such as ice water (15 %, *w*/w), salt (2 %, w/w), pepper (1 %, w/w), sodium tripolyphosphate (0.25 %, w/w) and ascorbic acid (0.15 %, w/w) were added. The minced meat was ground at low speed for 10 min and packed into the prepared casings, steamed at 85 °C for 60 min, dried at 45 °C for 30 min, and cooled to room temperature. The resulting sausages were immediately used for sensory evaluation and other experiments.

#### 2.15.2 Sensory evaluation of minced meat sausage

The test method was based on the method by Zhang et al. (2025) with minor modifications. The sensory evaluation panel consisted of 20 food science students with professional experience in sensory analysis. Members of the evaluation panel should be accustomed to consuming pork products at least twice a week and preferred traditional pork sausages. This study adopted the descriptive analysis method for sensory evaluation. Each panel member tasted different samples in turn and scored them according to various attributed of the samples, and 15 of the scoring sheets were randomly taken and analyzed to reduce the influence of subjective evaluation of the panel members on the experiment. The evaluation criteria and scores were shown in **Table S1**.

#### 2.15.3 Whiteness of minced meat sausage

The experimental methods were derived from published work (Zhang et al., 2024). The chromaticity of the finished sausages was measured using a CR-400 colorimeter (Minolta, Tokyo, Japan). The values of L*, a*, and b* were recorded, and the whiteness value of the finished sausage was evaluated by the following formula.

(11) $$\text{Whiteness}=100-\sqrt{{\left(100-L*\right)}^{2}-{\left(a*\right)}^{2}+{\left(b*\right)}^{2}}$$

### 2.16 Statistical analysis

All experiments were conducted independently by the authors of this work, where each experiment was guaranteed to be repeated at least three times to eliminate chance in the data. Experimental results were analyzed by SPSS 26.0 (IBM, NY, USA) using one-way analysis of variance (ANOVA) with Duncan's mean to compare the correlation between data (*p* < 0.05). Data graphs in this work were obtained after processing by Origin 2025SR1 (Origin Lab, MA, USA).

## 3 Results and discussion

### 3.1 Molecular docking simulation of PC-EWP

**Fig. 1:**
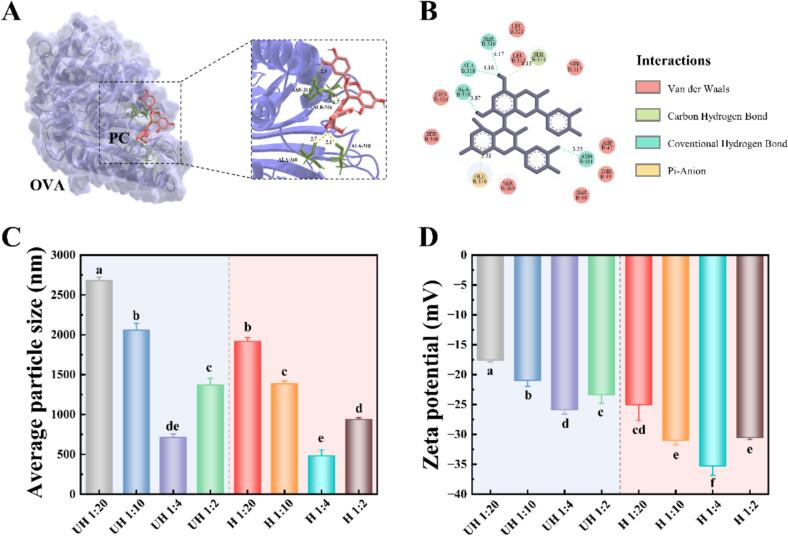
Three-dimensional schematic (A) and two-dimensional schematic (B) of PC-OVA molecular docking, and average particle size (C) and zeta potential (D) of PC-EWP emulsions as affected by heating and polyphenol content.

To elucidate the interaction mechanism between PC and EWP, this study selected ovalbumin (OVA), the most abundant protein in egg white, as the research subject. Utilizing its well-characterized three-dimensional structure, molecular docking simulations were employed to computationally model the binding modes between the two molecules. PyMOL-based three-dimensional visualization (Fig. 1A) demonstrated that PC molecules were embedded within a hydrophobic pocket on the surface of OVA, located in its C-terminal domain. This binding site was flanked by multiple hydrophobic residues (e.g., AlA-310, AlA-318) and polar residues (e.g., ASN-311, SER-316), suggesting that the interaction was likely mediated by synergistic hydrophobic forces and electrostatic complementarity. The phenolic hydroxyl groups of PC were oriented toward polar residues of OVA, forming a hydrogen-bond network (distance <3.0 Å) that stabilized the complex. The two-dimensional interaction diagram (Fig. 1B) further revealed robust hydrogen bonds between PC and ASN-311, SER-313, and SER-316, identifying these residues as critical determinants of binding specificity. Furthermore, hydrophobic residues (e.g., LEU-312, LEU-321) extensively enveloped the flavan backbone of PC through van der Waals interactions. These combined forces likely governed the initial ligand recognition and binding orientation, a finding consistent with prior simulation studies (Wen et al., 2023) This experimental work elucidated the molecular interaction mechanism between PC and OVA, providing a structural foundation for subsequent investigations into functional modulation mechanisms.

### 3.2 Particle size and zeta potential of emulsion

Building upon the molecular docking-revealed binding mode between PC and OVA, their complex formed via hydrogen bonds and hydrophobic interactions might significantly modulate protein surface charge distribution and spatial conformation, which directly influenced the interfacial behavior of proteins during emulsion formation (Yan et al., 2025). To further investigate this, the effects of heating treatment and PC concentration on the particle size (Fig. 1C) and zeta potential (Fig. 1D) of PC-EWP emulsions were systematically examined.

Notably, at a 1:4 ratio, PC achieved saturated binding with heated OVA via multisite hydrogen bonding and hydrophobic interactions, fully covering the oil-water interface to form a dense adsorption layer, thereby minimizing particle size (481 nm). However, excess PC tended to promote self-aggregation of the complex through π-π stacking, increasing particle size (Wen et al., 2023). Simultaneously, competitive adsorption of free polyphenols reduced the absolute zeta potential, weakening electrostatic repulsion. In summary, the synergistic regulation of heating and PC concentration enabled the formation of small, stable emulsion systems by modulating protein conformation and ligand binding saturation (Yu et al., 2025). Subsequent experiments on emulsion characteristics and surface tension would further validate their stability and functional properties.

### 3.3 Emulsification properties of emulsion

**Fig. 2:**
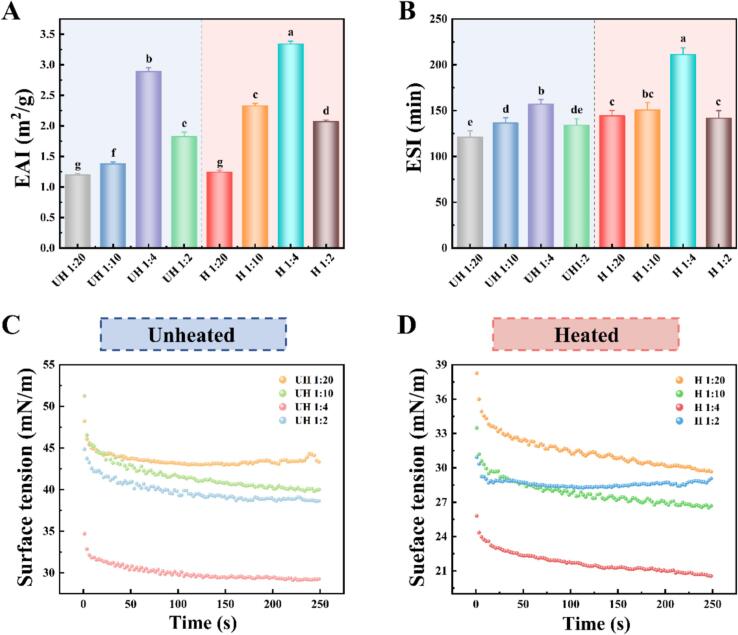
Emulsifying capacity (A), emulsion stability (B), and surface tension (C and D) of PC-EWP emulsions as affected by heating and polyphenol content.

As shown in Fig. 2A and B, heating significantly improved the emulsifying activity index (EAI) and emulsion stability index (ESI) of PC-EWP emulsions. This enhancement was attributed to partial protein denaturation induced by heating, which exposed hydrophobic residues and flexible regions, thereby strengthening hydrophobic synergism with the flavan backbone of PC and facilitating the formation of a more efficient and compact interfacial adsorption layer (Jin et al., 2025). At low PC concentration, unsaturated hydrogen-bonding sites limited protein adsorption and resulted in a discontinuous interfacial film, leading to reduced EAI and ESI values. Notably, under heated conditions at a 1:4 ratio, the emulsion achieved peak EAI and ESI values of 3.34 ± 0.04 m^2^/g and 211.28 ± 7.17 min, respectively. In this state, PC acted as molecular bridges, interconnecting multiple protein molecules to form a dense yet flexible interfacial network. The small particle size and enhanced cohesive viscosity reduced phase-separation kinetics, enabling resistance to environmental perturbations (Wen et al., 2023; Yan, Liu, Ren, et al., 2023). However, excessive PC triggered self-aggregation via π-π stacking, competitively occupying interfacial sites and diminishing EAI and ESI. Under such conditions, phase separation between free polyphenols and protein complexes created interfacial heterogeneity, while insufficient rigidity of the adsorption film accelerated droplet coalescence.

### 3.4 Dynamic surface tension of emulsion

Dynamic surface tension (DST) is a critical determinant of emulsification capacity (Yan et al., 2025). The DST profile can be divided into three phases: rapid adsorption, interfacial reorganization, and equilibrium stabilization. As can be seen from Fig. 2C, the unheated group exhibited a low adsorption rate, attributable to the restricted conformational flexibility of native OVA, which limited the accessibility of PC-binding sites and thereby impeded complex diffusion. Upon reaching equilibrium, this group displayed high equilibrium tension due to sparse interfacial films failing to effectively reduce surface energy. In contrast, heating accelerated adsorption (Fig. 2D), as exposed functional groups from denatured proteins rapidly anchored to PC, stabilizing the system via hydrogen bonding and hydrophobic cross-linking to form a dense interfacial film with lower equilibrium tension. Remarkably, the emulsion at a 1:4 ratio achieved the lowest equilibrium tension. Here, dynamic hydrogen bonding and a rigid hydrophobic network synergistically created a low-permeability interfacial layer, effectively suppressing Ostwald ripening (Zembyla et al., 2020). However, excessive PC competitively occupied interfacial sites with denatured proteins, compromising membrane continuity and increasing equilibrium tension, consistent with the observed decline in ESI. In summary, DST analysis demonstrated that the synergistic effects of thermal treatment and PC concentration governed emulsion performance by modulating interfacial adsorption kinetics and membrane stability.

### 3.5 The double network emulsion gel (DNEG) texture characterization

**Fig. 3:**
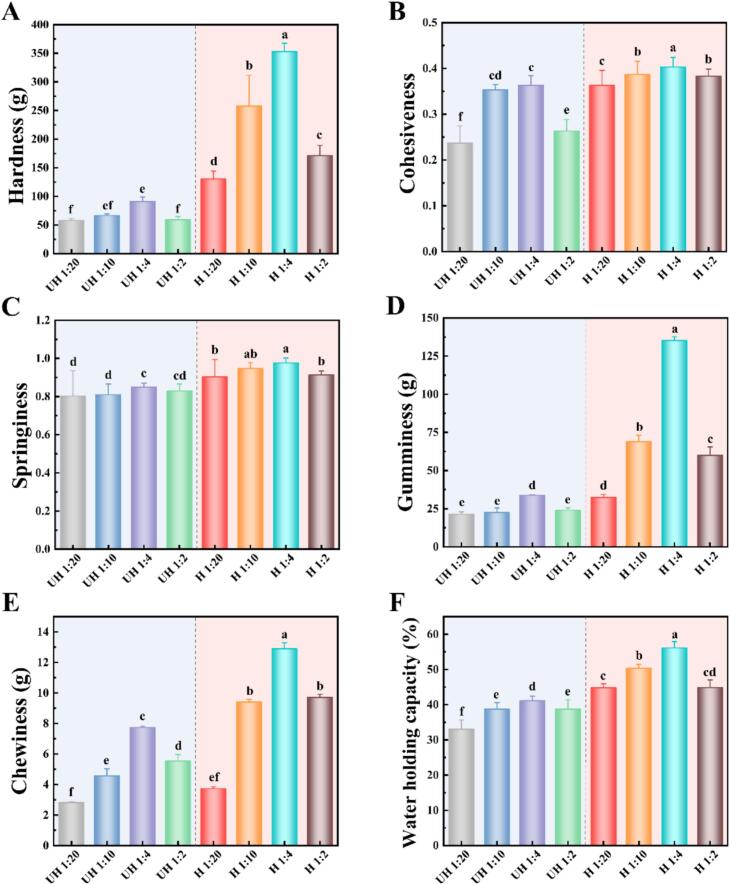
Textural (Hardness (A), Cohesiveness (B), Springiness (C), Gumminess (D), Chewiness (E)) and water-holding properties (F) of DNEG with different heating and PC contents.

Subsequently, the DNEG were fabricated using PC-EWP emulsions and its structural properties were characterized. TPA served as a critical method to evaluate the oral sensory characteristics and textural properties of the emulsion gel-based fat substitutes (Xu et al., 2024). As illustrated in Fig. 3A, the hardness of the unheated (UH) group was significantly lower than that of the heated (H) group. This discrepancy arose because the stable hierarchical structure of “globular proteins” in the absence of appropriate thermal treatment hindered deep intermolecular cross-linking among EWPs, thereby severely restricting polyphenol-protein interactions, which was consistent with prior conclusions (Zhang et al., 2023). It had been emphasized that heat-treated proteins formed distinct and dense three-dimensional networks during acid-induced gelation, significantly enhancing the hardness of high internal phase Pickering emulsion gels, as demonstrated in coix seed oil-based systems (Li, Li, Wu, et al., 2025). Cohesiveness (Fig. 3B), a parameter reflecting internal binding strength, reached its peak (0.403) at a ratio of 1:4. This improvement was attributed to the formation of PC-EWP conjugates, which reduced emulsion droplet size and suppress flocculation and coagulation during acid-induced gelation, as previously discussed. The diminished interfacial tension weakened internal repulsive forces during gel formation, thereby enhancing aggregation density. Similarly, the springiness (Fig. 3C) and gumminess (Fig. 3D) of DNEG exhibited analogous trends, originating from variations in the compactness of the gel network. In addition, the increase in elasticity was also due to the reduction in interfacial tension and emulsion droplet size mediated by polyphenols, thereby enhancing network continuity and mechanical strength (Yin et al., 2023). Increasing polyphenol content introduced additional phenolic hydroxyl groups, which refined the double network architecture with small pore sizes (Zhang et al., 2025). Consequently, the chewiness (Fig. 3E) of DNEG modified by combined thermal and polyphenol treatments significantly surpassed that of the control group, which enhanced their potential as fat substitutes for improved oral perception.

### 3.6 DNEG water holding properties (WHC)

Notably, heating treatment markedly elevated the WHC of DNEG (Fig. 3F). This enhancement stemmed from partial heat-induced denaturation of EWP, which exposed buried hydrophobic domains and free sulfhydryl groups (Zhang et al., 2025). The unfolded protein molecules formed a more continuous and ordered three-dimensional network through strengthened hydrophobic interactions and disulfide bonds. This robust network physically restricted water migration through physical sequestration and capillary action (Gao et al., 2024). In contrast, untreated EWP gels exhibited weaker water-binding capacity due to insufficient cross-linking density. The divergent performance of DNEG underscored the dynamic restructuring of intermolecular forces, necessitating further exploration of synergistic regulatory mechanisms among key interactions within the gel network.

### 3.7 Intermolecular forces in DNEG

**Fig. 4:**
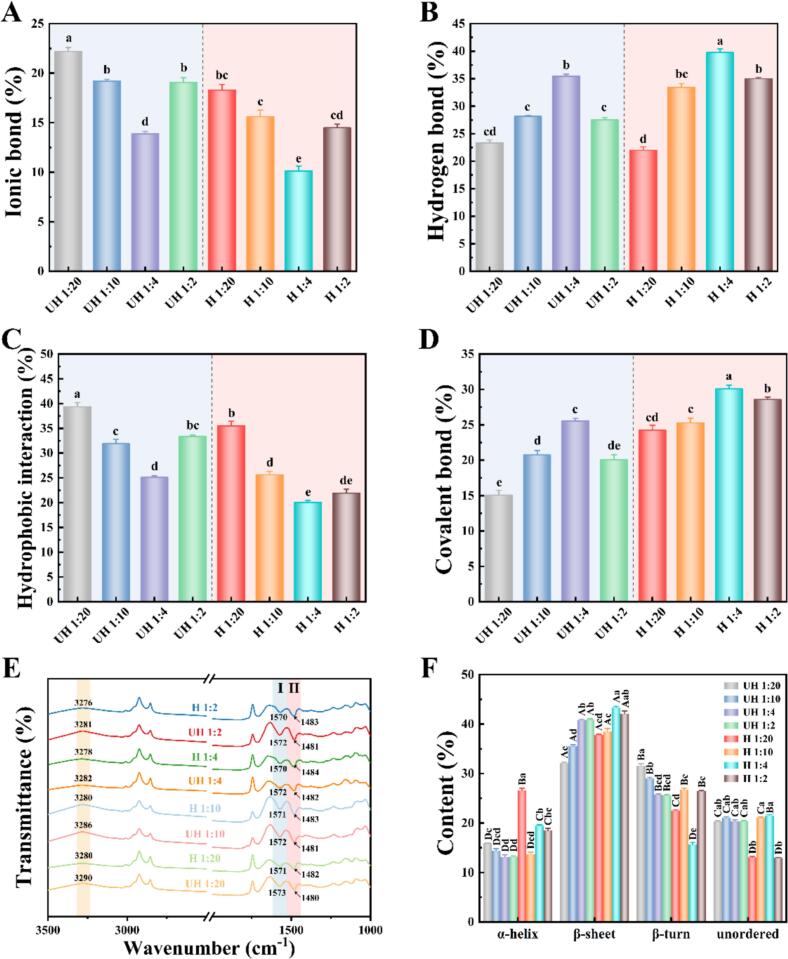
Intermolecular interaction forces (Ionic bond (A), Hydrogen bond (B), Hydrophobic interaction (C), Covalent bond (D)), infrared spectra (E), and secondary structure content (F) of DNEG as affected by heating and PC content.

The structural changes in the emulsion gel network originated from differences in intermolecular interactions (Deng et al., 2020). As demonstrated in Fig. 4A, heat treatment significantly reduced the contribution of ionic bonds, which might be associated with the unfolding of EWP and deprotonation of charged residues. The addition of PC presented a concentration-dependent effect on the proportion of ionic bonds. At low PC ratios (1:20–1:10), the impact on ionic bonds was minimal. However, at a higher ratio (1:2), PC significantly weakened ionic bonds by competitively adsorbing and shielding protein charges. This was attributed to excessive phenolic hydroxyl groups modulating ionic bond strength through altered protein surface charge distribution (Wu et al., 2024). When EWP was subjected to heat treatment, its exposed hydrophobic domains markedly enhanced hydrophobic interactions, while the introduction of PC further restructured the force balance (Fig. 4B). At a 1:4 ratio, polyphenols bound to the hydrophobic regions of heat-denatured proteins, forming stable hydrophobic crosslinks. Excessive PC induced nonspecific aggregation, thereby disrupting the cooperativity of hydrophobic interactions. Furthermore, as revealed in Fig. 4C, increasing the PC ratio promoted the formation of dense hydrogen bonds between phenolic hydroxyl groups and polar residues of EWP, which partially compensated for hydrogen bond loss caused by preheating. Remarkably, covalent bond alterations were closely correlated with both heat treatment and polyphenol addition (Fig. 4D). Heating treatment significantly promoted disulfide bond formation by activating free sulfhydryl groups in protein (Tang et al., 2011). At a 1:4 ratio, PC not only protected sulfhydryl groups from excessive oxidation but also stabilized crosslinked structures through hydrogen bonds, maximizing covalent bond contribution, which revealed the reason for the enhanced gel hardness as described in Section 3.5. In contrast, high PC concentrations (1:2) hindered sulfhydryl crosslinking via steric hindrance, reducing disulfide bond proportions and further validating the dynamic equilibrium between covalent and non-covalent interactions. These results demonstrated that the PC/EWP ratios and heat treatment have a synergistic effect on EWP. This would be beneficial for the targeted regulation of intermolecular forces in the gel network and the reshaping of its macroscopic properties.

### 3.8 DNEG secondary structure

FT-IR was employed to analyze amide bands, revealing the regulatory mechanisms of protein secondary structures (Li, Luo, Zhang, et al., 2025). As illustrated in Fig. 4E, heat treatment combined with polyphenols induced amide band shifts. When the PC/EWP ratio increased from 1:20 to 1:4, the amide I band blue-shifted from 1573 cm^−1^ (unheated) or 1570 cm^−1^ (heated) to 1642 cm^−1^. This indicated that phenolic hydroxyl groups of PC formed new hydrogen bonds with protein C=O groups, which promoted β-sheet conformations (Zhang et al., 2025). Additionally, the amide A band exhibited continuous red shifts (3290 cm^−1^ to 3276 cm^−1^) with increasing PC ratios, further confirming the regulatory role of polyphenol hydroxyl content on hydrogen bond strength, which arose from interactions between phenolic hydroxyls (PC) and amide bonds (EWP).

Further, by inverse convolution fitting (Fig. 4F), it was found that PC addition with preheating treatment significantly changed the secondary structure occupancy by analyzing the results. Heat treatment reduced α-helix content while markedly increasing β-sheets and random coils. This phenomenon was attributed to PC anchoring denatured protein chains into β-sheets via dense hydrogen bonding, thereby suppressing disordered aggregation. Excessive PC elevated β-turn proportions by 40.9 % and decreased random coil content by 39.5 %. Moderate random coils likely enhanced water holding capacity through increased network porosity, but excessive disorder (1:2 ratio) caused structural collapse (Chen et al., 2025). The reorganization of secondary structures not only governed molecular conformational dynamics of the gel but also shaped its microscopic morphology by modulating protein chain alignment. Therefore, further clarification was needed by observing the microstructure of the gel.

### 3.9 Microscopic morphology of DNEG

**Fig. 5:**
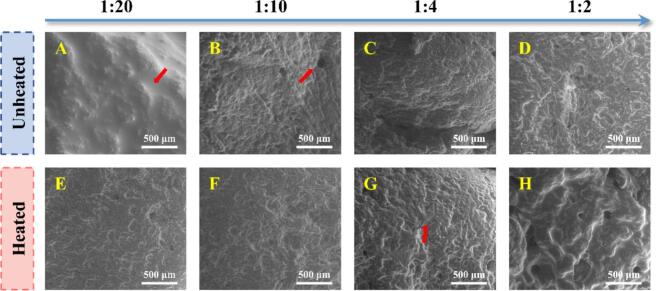
Microstructural characterization of DNEG (SEM images).

SEM was utilized to evaluate the microstructure of gels, which provided insights into the application potential of fat substitutes (Du & Meng, 2025). The synergistic regulation of heat treatment and PC on molecular chain crosslinking significantly influenced the pore structure distribution of the DNEG (Fig. 5). Untreated samples exhibited heterogeneous structures with thin pore walls and oil phase leakage (Fig. 5A). This morphology originated from the crosslinking dominated by the globular conformation of native EWP, which relied on ionic bonds and weak hydrogen bonds, leading to insufficient protein chain aggregation and low mechanical strength of the network (Ma et al., 2023). Heat-induced protein denaturation markedly improved network homogeneity and thickened pore walls (Fig. 5E). These enhancements arose from strengthened hydrophobic interactions and disulfide bonds, promoting the formation of a dense and coherent three-dimensional network, which significantly enhanced structural stability and water holding capacity as mentioned in Section 3.6. With increasing polyphenol content, the microstructure gradually evolved into a highly uniform honeycomb-like porous structure (Fig. 5G), indicating that the synergy between polyphenols and heat treatment balanced the mechanical strength and ductility of DNEG. However, excessive PC addition resulted in flocculent aggregates with enlarged pore sizes (Fig. 5H), attributed to reduced flexibility caused by over-interactions between phenolic hydroxyl groups and proteins (Zhang et al., 2025). The directional design of microstructural morphology established a foundation for developing emulsion gel-based fat substitutes with high water retention and controllable texture, which inevitably influenced the digestive behavior of DNEG.

### 3.10 DNEG digestive properties

#### 3.10.1 Digestive properties

**Fig. 6:**
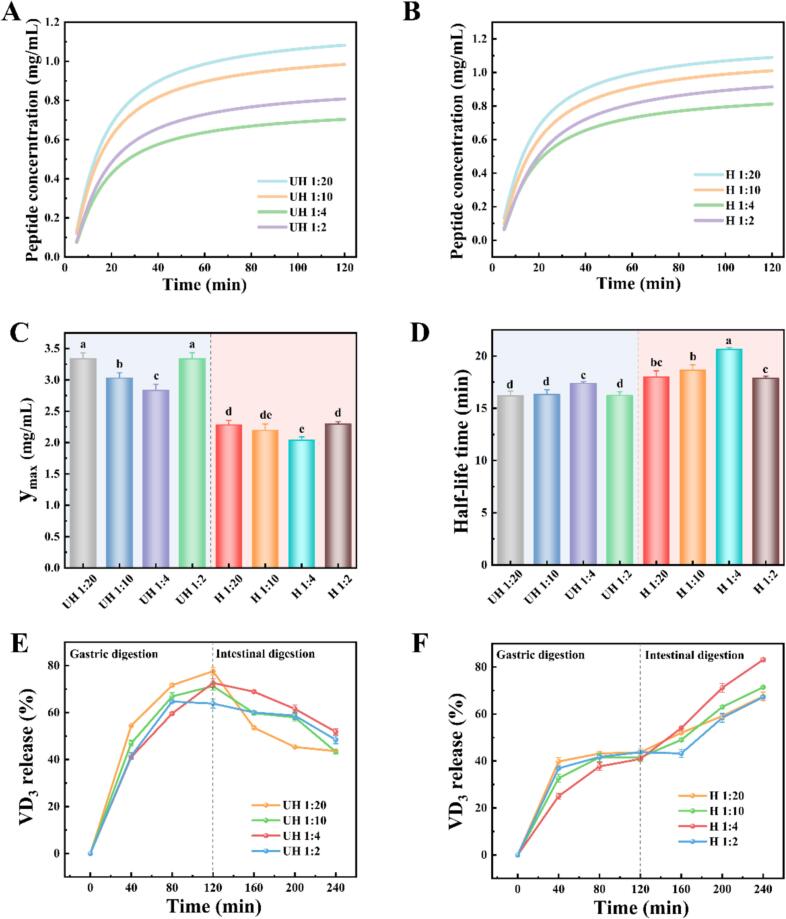
Fitted curves of digestion kinetics of DNEG as affected by heating and PC content (A and B), maximum peptide concentration (y_max_ (C)), half-life (D), and release of vitamin D_3_ (E and F).

**Fig. 7:**
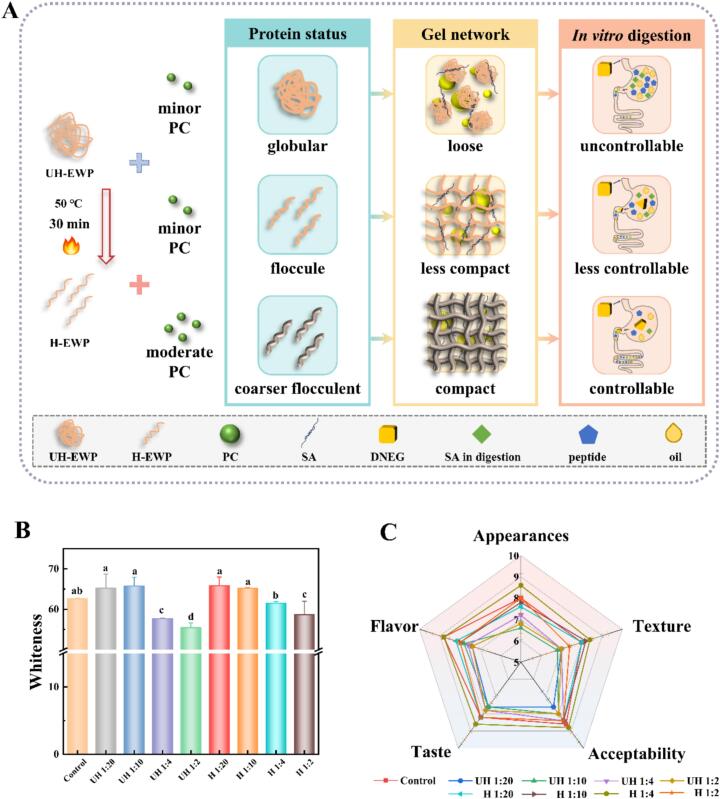
Diagram of the structure and digestion mechanism of DNEG as affected by heating and PC content (A), whiteness (B), and sensory evaluation (C) of minced meat sausages.

To elucidate the sequential changes of emulsion gel-based fat substitutes during digestion, their digestive kinetics were analyzed in detail using established methodologies (Liu et al., 2023). The first-order kinetic model fitting of DNEG in simulated gastric conditions revealed that untreated samples exhibited fast digestion rates (Fig. 6A), indicating rapid protein hydrolysis without controlled-release capability. This was attributed to the loose gel network formed by native EWP, which exhibited poor enzymatic resistance. In contrast, heat-treated gels demonstrated slower digestion rates upon polyphenol incorporation (Fig. 6B). The hydrophobic binding between PC and heat-denatured EWP improved the gel matrix, synergistically reducing DNEG pore size and prolonging pepsin diffusion pathways. Concurrently, the y_max_ value of the sample decreased, and peptide release correspondingly decreased as indicated in Fig. 6C. Furthermore, the half-life extended to 20.7 min, representing a 28 % increase over the untreated group (Fig. 6D), which was ascribed to enhanced covalent interactions within the gel matrix reinforced by PC, thereby decelerating gel disintegration (Molet-Rodríguez et al., 2023). The synergistic effects of polyphenols and thermal treatment enabled effective control of digestion rates by optimizing network compactness and enzymatic resistance. (See Fig. 7.)

#### 3.10.2 Vitamin D_3_ release characteristic

To evaluate the sustained-release capacity of DNEG for bioactive compounds, vitamin D_3_ (VD_3_) was encapsulated, and its release characteristic was assessed (Fig. 6E-F). It was found that VD_3_ in untreated samples was predominantly released during the gastric phase (release rate > 65 %), as gastric acid and digestive enzymes substantially weakened the gel's crosslinking effects. Prematurely released VD_3_ underwent partial degradation in the acidic and oxidative gastric environment, while the structurally compromised residual gel failed to achieve controlled intestinal release, hindering bioactive delivery to the intestinal site (Yang et al., 2020). Notably, VD_3_ release in the intestinal phase was significantly enhanced in heat-treated gels, with the H 1:4 group achieving total VD_3_ release of 83.1 %. This improvement was attributed to PC-enhanced network compactness in EWP-based DNEG, which stabilized VD_3_ and enabled targeted intestinal release. The dense network induced by heat treatment inhibited permeation of gastric acid and proteases, while optimal PC concentrations improved VD_3_ stability through micellization regulation (Li, Li, Li, et al., 2025). These findings provided novel insights for developing digestion-controllable emulsion gel fat substitutes, which necessitated further mechanistic hypotheses to enhance the reference value of this work.

### 3.11 Mechanism of controlled DNEG digestion

Based on the multidimensional characterization of DNEG, their digestion-controllable mechanism was rationally hypothesized. Untreated EWP retained its native globular conformation, primarily relying on ionic bonds and weak hydrogen bonds for intermolecular crosslinking. Low polyphenol concentrations (1:20) supplemented limited hydrogen bonds via surface adsorption, forming a loose porous network. This allowed digestive enzymes (e.g., pepsin, trypsin) to rapidly diffuse through large pores in the coarse network during digestion, which led to swift hydrolysis of EWP. Heat-induced denaturation unfolded EWP, forming a moderately dense network dominated by hydrophobic interactions and disulfide bonds. However, insufficient polyphenol addition failed to establish a dense continuous barrier, diluting enzyme-inhibitory activity. Consequently, this system neither delayed DNEG digestion nor achieved sustained or targeted release functionality. It was noteworthy that hydrophobic domains exposed by heat-denatured EWP interacted with polyphenols to form high-strength protein architectures. This facilitated the development of compact DNEG networks, significantly prolonging digestive enzyme diffusion pathways. Heating established the structural foundation of the network, while optimal polyphenol concentrations enabled precise regulation of digestion kinetics through covalent/non-covalent crosslinking and enzyme inhibition. This mechanism provided a theoretical framework for developing intelligent-responsive fat substitutes, which expanded the application potential of EWP-based emulsion gels in functional foods.

### 3.12 Sensory evaluation of low-fat sausages

The use of fat substitutes inevitably exerted significant impacts on the visual appeal, textural properties, and overall acceptability of low-fat products, which prompted systematic evaluations of low-fat sausages (Lu et al., 2024). Sausages prepared with untreated DNEG as fat replacements exhibited soft texture, insufficient elasticity, and low sensory scores for appearance and texture. This was likely caused by weak gel water-holding capacity, leading to juice leakage. Compared to the control group, heat-treated samples provided more effective support for the meat gel network as fat mimetics, significantly increasing taste scores. The synergistic effects of polyphenol incorporation and heat treatment enabled the PC: EWP = 1:4 sausage samples to achieve flavor, appearance, and overall acceptability comparable to full-fat sausages. This improvement was attributed to phenolic hydroxyl groups of PC stabilizing the gel structure via hydrogen bonds, thereby entrapping moisture and flavor compounds within the meat gel matrix through capillary forces (Zhang et al., 2025).

### 3.13 Coloration of low-fat sausages

To comprehensively evaluate product quality, the color properties of the sausages were further analyzed. Whiteness values across all systems gradually decreased with increasing PC ratios, as the inherent reddish-brown hue of PC significantly masked whiteness (Zhang et al., 2024). However, preheating mitigated color interference by optimizing network homogeneity, slowing the decline in whiteness compared to the control. This phenomenon likely arose from enhanced light reflection by uniform micropores, compensating for light absorption by PC and aligning whiteness with full-fat products. These findings established a theoretical foundation for designing EWP-based fat substitutes with balanced functional and sensory properties.

## 4 Conclusion

This study systematically elucidated the mechanism by which PC-assisted thermal modification of EWP regulated DNEG structural evolution and digestion properties. Thermal treatment induced EWP conformational unfolding, enabling exposed hydrophobic groups to form robust hydrophobic crosslinks with PC. This significantly optimized interfacial tension, promoting the formation of dense and homogeneous gel networks. The enhanced textural properties of DNEG originated from synergistic molecular force reconstruction: hydrophobic interactions and disulfide bonds dominated the crosslinked framework, while dynamic hydrogen bonding via PC's phenolic hydroxyl groups enhanced network flexibility, which created porous structures with balanced mechanical strength and water-holding capacity. Microstructural analysis further revealed that PC-thermal synergy induced a highly ordered dense network, effectively improving structural stability. Digestion kinetics demonstrated that the compact DNEG network prolonged enzymatic diffusion pathways, enabling sustained VD_3_ release. As a fat substitute, DNEG exhibited sensory characteristics comparable to full-fat counterparts in low-fat systems, with functional superiority attributed to dynamic force equilibria and precision microstructural modulation. This work provided theoretical and technical foundations for designing protein-based gels via thermal-polyphenol synergy and advancing their application in digestion-controllable fat substitutes.

## CRediT authorship contribution statement

**Renzhao Zhang:** Writing – review & editing, Writing – original draft, Methodology, Funding acquisition. **Jingbo Liu:** Supervision. **Meijing Yu:** Investigation. **Qiri Mu:** Conceptualization. **Zhaohui Yan:** Writing – review & editing, Funding acquisition, Data curation. **Yudan Zhang:** Formal analysis. **Yutong Zhang:** Investigation. **Ting Zhang:** Supervision. **Xuanting Liu:** Writing – review & editing, Supervision, Funding acquisition.

## Declaration of competing interest

The authors declare that they have no known competing financial interests or personal relationships that could have appeared to influence the work reported in this paper.

## Data Availability

Data will be made available on request.

## Appendix A Supplementary data

Supplementary material Image 1
