# Supplementary material for: Emerging strategies for controlled digestion of fat substitutes: Synergistic modification of egg white protein by combined polyphenol heat treatment for preparation of double network emulsion gel
Source: Food Chem X. 2025 Jul 12;29:102784. doi: 10.1016/j.fochx.2025.102784 (PMC12281071; doi:10.1016/j.fochx.2025.102784)
Supplement: Supplementary file 1 — Supplementary material [file mmc1.docx]

**Emerging strategies for controlled digestion of fat substitutes: Synergistic modification of egg white protein by combined polyphenol heat treatment for preparation of double network emulsion gel**

Renzhao Zhang^1^, Jingbo Liu^1^, Meijing Yu^1^, Qiri Mu^1^, Zhaohui Yan^1^, Yudan Zhang^1^, Yutong Zhang^1^, Ting Zhang^1^, Xuanting Liu^1*^

^1^: Jilin Provincial Key Laboratory of Nutrition and Functional Food and College of Food Science and Engineering, Jilin University, Changchun 130062, China.

*Corresponding Author: Xuanting Liu, Jilin Key Laboratory of Nutrition and Functional Food, Jilin University Changchun, 130062, PR. China.

E-mail: [lxt920523@163.com](mailto:lxt920523@163.com)

**Table S1. The sensory evaluation scoring criteria of minced meat sausages.**

| **Sensory properties** | **Definition of assessment terms** | | |
| --- | --- | --- | --- |
|  | **1~3** | **4~6** | **7~10** |
| **Color** | Uneven color distribution, obvious graininess, poor luster | More uniform color distribution, fewer particles, more even luster | Uniform color distribution, no obvious graininess, glossy |
| **Texture** | Loose slices, rough surfaces, very heterogeneous porosity | The slices are a bit loose, the surface is not very smooth and there are visible air holes on the surface | Firmly sliced, smooth surface, uniform air holes |
| **Acceptability** | bad reaction | general reaction | good reaction |
| **Taste** | Meat rough, uneven, hard, poor elasticity, bad-greased | Delicate, uneven, firm flesh, moderate grease | Delicate, even, full-fleshed and well-greased |
| **Flavor** | No meaty taste or odor | Not enough meat flavor, slightly off-flavor | Strong meat flavor, no off-flavors |
